# Assessing Variability in Reporting Severity of the Same Symptom (Fatigue) in the Context of Different Psychiatric Syndromes

**DOI:** 10.7759/cureus.44112

**Published:** 2023-08-25

**Authors:** Maya Subramanian, Timothy McAuliffe, Himanshu Agrawal

**Affiliations:** 1 College of Medicine, Medical College of Wisconsin, Milwaukee, USA; 2 Psychiatry and Behavioral Medicine, Medical College of Wisconsin, Milwaukee, USA; 3 Psychiatry, Medical College of Wisconsin, Milwaukee, USA

**Keywords:** psychiatry and mental health, clinical psychiatry, fatigue scales, general anxiety disorder, major depression disorder

## Abstract

Objective

The objective of this study is to examine the variability in the self-reported fatigue symptom severity in major depressive disorder (MDD) compared to generalized anxiety disorder (GAD).

Methods

A retrospective chart review was conducted of 100 patients evaluated for fatigue using depression and anxiety questionnaires. The study examined whether ratings of fatigue varied based on whether fatigue was being rated by the patient in the context of MDD vs. when fatigue was being rated by the same patient in the context of GAD. A related-sample Wilcoxon signed-rank test and Mann-Whitney U test were used to compare the median differences between depression and anxiety fatigue scores. The significance level used was 0.05.

Results

This study found a statistically significant difference in the median difference of the paired depression fatigue and anxiety fatigue scores (depression score - anxiety score) regardless of the order of administration (Wilcoxon signed-rank test statistic = 135.500, p-value =.008, N = 100 paired scores).

Conclusion

The study's conclusions show that although the symptom of fatigue is listed in the Diagnostic Statistical Manual 5 (DSM-5) criteria for MDD as well as GAD, it may be perceived by patients differently based on the context of the syndrome. This emphasizes the importance of considering the context of symptom reporting in patients with MDD and GAD to improve diagnostic methodologies and treatment strategies.

## Introduction

Fatigue is a common symptom experienced by individuals with both major depressive disorder (MDD) and generalized anxiety disorder (GAD) [1]. Fatigue is defined as a persistent state of physical and mental weariness, leading to a profound sense of exhaustion and diminished overall functioning [2]. Fatigue is included in the diagnostic criteria for both disorders in the Diagnostic and Statistical Manual of Mental Disorders (DSM-5) as well as in its most recent variant, the Diagnostic and Statistical Manual of Mental Disorders, Fifth Edition, Text Revision (DSM-5-TR) [3]. However, there may be variations in the way patients self-report the symptom of fatigue depending on which disorder is being assessed for.

Depression is a mental health disorder characterized by persistent low mood, loss of interest in normally enjoyable activities, and a range of physical and emotional symptoms, such as fatigue, sleep disturbances, and feelings of worthlessness. Anxiety is characterized by excessive fear, worry, and nervousness, often in the absence of a reasonable cause (DSM-5). Depression and anxiety are often comorbid and can have similar or overlapping symptomatic presentations [3]. For example, fatigue is a common symptom experienced by individuals with both anxiety and depression [4]. Despite the shared presentation of fatigue in these two syndromes, the way in which it is reported by patients differs depending on the context (the syndrome) in which it is experienced.

Several studies have explored the differences in the way patients report fatigue in the context of anxiety and depression. A study by Dialogues in Clinical Neuroscience found that patients with depression reported higher levels of fatigue compared to those with anxiety. Furthermore, patients with depression reported that their fatigue was more persistent and detrimental to their daily activities compared to those with anxiety [5]. In addition, patients with depression reported more physical symptoms associated with their fatigue, such as headaches and muscle pain, compared to those with anxiety [6].

On the other hand, a study in the Journal of Anxiety Disorders found that patients with anxiety reported more severe and persistent fatigue compared to those with depression. Additionally, patients with anxiety reported that their fatigue was more closely associated with their psychological symptoms, such as worry and nervousness, compared to those with depression [7].

Literature suggests that the way in which patients experience and report fatigue may vary depending on whether it is in the context of anxiety or depression. While patients with depression tend to report higher levels of fatigue and physical symptoms associated with it, those with anxiety report fatigue that is closely related to their psychological symptoms [8]. It is important to consider the context in which fatigue is experienced when evaluating and treating patients with psychiatric disorders. Understanding the differences in the way patients report fatigue in the context of anxiety and depression can help healthcare providers provide more accurate and effective treatment for their patients.

There is a gap in the literature about the context in which individuals self-report their symptoms, specifically regarding major depressive disorder (MDD) and generalized anxiety disorder (GAD). While it is known that fatigue is a common symptom in both disorders, the way in which individuals experience and report it may differ depending on the context in which it is assessed. This gap in the literature makes it difficult for healthcare professionals to accurately assess and diagnose patients as well as provide effective treatment. This study emphasizes the need for future research to delve deeper into symptom overlap while also extending investigations to other psychiatric disorders with shared symptoms. The prospect of a comprehensive exploration of commonalities among anxiety, depression, and post-traumatic stress disorder (PTSD) symptoms presents an important avenue for research.

This research is necessary to help fill this gap in the literature and to provide more information about the way in which individuals self-report their symptoms depending on the context in which they are asked. This information can be used by healthcare professionals to develop more accurate diagnostic tools and provide more effective treatment for individuals with MDD and GAD. Additionally, this research can help shed light on the relationship between MDD and GAD and the potential impact of the context in which symptoms are assessed on the accuracy of diagnosis and treatment. By understanding the relationship between context and symptom self-reporting, healthcare professionals can provide more comprehensive and effective care for individuals with these psychiatric disorders.

This gap in the literature is significant, as the order of questionnaire administration may influence how individuals perceive and self-rate their symptoms. For example, if depression is assessed first, it may impact the subsequent assessment of anxiety, and vice versa. Additionally, the way in which depression and anxiety are perceived and self-rated may vary based on the individual's personal experiences and perception of these conditions. As a result, it is important to consider the order in which depression and anxiety are assessed to ensure that the results accurately reflect the individual's experiences and to minimize any potential biases in the assessment process. Further research is needed to better understand the impact of the order of questionnaire administration on the perception and self-rating of depression and anxiety.

## Materials and methods

This study involved a retrospective chart review of 100 patients who had been diagnosed with MDD as well as GAD (based on DSM-5 criteria) at the time of the visit. Written informed consent was waived by the institution's IRB since it would have been unfeasible to do so in this retrospective chart review (per Section 164.502(b) of the Privacy Rule under federal regulations). All ethical considerations were meticulously followed, guided by the protocols and policies established by the institute's Institutional Review Board (IRB). Before initiating the study, formal ethical approval was obtained from the IRB. All these patients had been seen by one of the authors (Himanshu Agrawal) in his outpatient practice. The study analyzed data from 100 patients seen at the principal investigator’s (PI's) Medical College of Wisconsin (MCW) Outpatient Psychiatry Clinic at the Tosa Health Center between July 1st, 2017, and June 30th, 2022. The inclusion criteria for this study were the principal investigator’s (Himanshu Agrawal) patients at his outpatient psychiatry practice, and the exclusion criteria were any patients who did not meet the inclusion criteria. Patients were evaluated for fatigue using a depression questionnaire and an anxiety questionnaire (Table 1). The questionnaires used in this study were developed by the research team, drawing on the diagnostic criteria outlined in the DSM-5-TR. The patients were asked to rate their symptoms of depression and anxiety individually from zero (not at all severe) to three (very severe). The study aimed to examine whether the ratings of fatigue differed based on which syndrome was asked about and if ratings varied based on which syndrome was asked about first (anxiety or depression).

**Table 1:** Depression and anxiety symptom severity table on average over the last two weeks (zero-three)

Depressive symptoms	Anxious symptoms
Little interest or pleasure in doing things	Feeling nervous, anxious, or on edge
Feeling down, depressed, or hopeless	Not being able to stop or control worrying
Trouble falling or staying asleep, or sleeping too much	Worrying too much about differential things
Feeling tired or having little energy	Trouble relaxing or feeling tense in the muscles
Poor appetite or overeating	Being so restless that it is hard to sit still
Feeling bad about yourself or that you are a failure or have let yourself or your family down	Feeling tired or having little energy
Trouble concentrating on things, such as reading the newspaper or watching television	Becoming easily annoyed or irritable
Moving or speaking so slowly that other people could have noticed. Or the opposite- being so fidgety or restless that you have been moving around a lot more than usual	Feeling afraid as if something awful might happen
Thoughts that you would be better off dead, or hurting yourself in some way	
TOTAL (27)	TOTAL (24)

To achieve this, the patients were divided into two groups (50 patients per group): within the first group, depression-related questions were asked first, and for the second group, anxiety-related questions were asked first. The depression-related scores among the two groups were compared using the independent-samples Mann-Whitney test to determine if the depression-related fatigue scores differed when asked first or last. The same process was repeated for the anxiety-related fatigue scores.

To investigate whether the ratings of fatigue differed based on which syndrome was asked about, the related-samples Wilcoxon signed-rank test was used to compare the paired depression and anxiety fatigue scores (a total of 100 patients). Lastly, the Mann-Whitney test was used to examine whether the order of administration impacted the difference between the depression fatigue and anxiety fatigue scores (depression score - anxiety score). All the statistical analyses were performed using a significance level of 0.05.

## Results

The investigation into the impact of the order of administration on fatigue scores, specifically in relation to the depression measure, revealed intriguing insights. The comparison of medians for the two groups, where the depression measure was administered first versus last, demonstrated no statistically significant difference (Mann-Whitney U statistic = 1064.5, p-value = 0.19). This statistical outcome indicates that the sequence in which the depression measure was administered did not yield discernible variations in the observed fatigue scores among the participants.

A parallel examination was conducted where the anxiety questionnaire was administered first. The comparison of medians between the group with the anxiety measure administered first and the group with it administered last revealed a non-significant outcome (Mann-Whitney U statistic = 1170.0, p-value = 0.57). These findings underscore that the order of administering the anxiety measure did not translate into meaningful differences in the reported fatigue scores.

Further exploration centered on the paired fatigue scores when the anxiety measure was administered first to a subset of 50 patients. In this scenario, the paired depression fatigue and anxiety fatigue scores demonstrated a statistically significant difference (Wilcoxon signed-rank test statistic = 148.0, p-value = 0.005). This indicates that the order of administration played a discernible role in shaping the perception of fatigue among these individuals. However, administering the depression measure first to a separate group of 50 patients did not yield a statistically significant difference in paired fatigue scores (Wilcoxon signed-rank test statistic = 74.0, p = 0.42).

The study extended its examination to compare the paired depression fatigue and anxiety fatigue scores among patients based on whether the depression measure was administered first or last. The analysis revealed no statistically significant difference between these two patient groups (Mann-Whitney U statistic = 1042.5, p-value = 0.09). This implies that the sequence of administration of the depression measure did not significantly contribute to divergences in fatigue scores.

Upon disregarding the order of administration of syndrome symptom measures and focusing on the entire patient sample (n = 100), a compelling observation emerged. The fatigue scores demonstrated a statistically significant difference (Wilcoxon signed-rank test statistic = 135.5, p-value = 0.008). This overarching trend suggests that the cumulative influence of both the depression and anxiety measures on fatigue scores became evident, transcending the specific order in which they were administered.

Table 2 shows the overall comparison of the fatigue scores by order of administration and the difference in the paired depression fatigue and anxiety fatigue scores (depression score - anxiety score). Individually, when the anxiety measure was administered first (to 50 patients), the paired depression fatigue and anxiety fatigue scores were found to differ statistically significantly (Wilcoxon signed-rank test statistic = 148.0, p-value =.005), but not when the depression measure was administered first (to 50 patients) (Wilcoxon signed-rank test statistic = 74.0, p =.42). When the difference between the paired depression fatigue and anxiety fatigue scores (depression score - anxiety score) among patient groups for whom the depression measure was administered first and last was compared, the groups were not found to be statistically significantly different (Mann-Whitney U statistic = 1042.5, p-value =.09). However, among the entire patient sample, when the order of administration of the syndrome symptom measures is ignored, the scores are found to differ statistically significantly (Wilcoxon signed-rank test statistic = 135.5, p-value =.008, with a sample of 100 paired scores).

**Table 2:** Comparison of fatigue scores by order of administration

Sample	n	Depression fatigue score (Mean (SD))	Anxiety fatigue score (Mean (SD))	Difference between fatigue score	p
Depression measure was administered first	50	1.71 (0.97)	1.79 (1.03)	-0.08 (0.53)	0.42
Anxiety measure was administered first	50	1.46 (0.97)	1.70 (0.91)	-0.24 (0.51)	0.005
Combined (with order of administration ignored)	100	1.58 (0.97)	1.74 (0.96)	-0.16 (0.52)	0.008

## Discussion

The findings of this study highlight the importance of considering the context in which symptoms are assessed when evaluating patients with comorbid MDD and GAD [9]. While the order of administration did not have a significant impact on self-reported fatigue scores in individuals with MDD or GAD, the study suggests that patients may perceive the same symptom, such as fatigue in this case, differently in the context of different psychiatric syndromes [10]. Specifically, the study found that individuals reported higher levels of fatigue when it was assessed in the context of their GAD compared to when the same symptom was assessed in the context of their MDD.

It is important to note that these findings are limited by the small sample size of the study, and further research with larger sample sizes would help to confirm these findings and better understand the large-scale impact of this study in the evaluation of individuals with MDD and GAD. Nonetheless, the present study displays the importance of considering the context in which symptoms are assessed when evaluating and treating patients with comorbid psychiatric conditions. The authors submit that mental health professionals should consider entire clinical scenarios when evaluating a psychiatric condition (in this case, MDD or GAD) and that any one symptom may not provide diagnostic clarity or reflect the severity of the disease (since the same question regarding fatigue was answered differently by the same patient when asked in different contexts) [11]. The study was also limited to individuals who met both the criteria for MDD and GAD. It would be crucial to see if the same findings are repeated when comparing individuals who meet criteria for only MDD vs. individuals who meet criteria for only GAD (not MDD). Next, the sample was limited to patients seen at a single clinic by a single clinician. It will be important to see if similar findings can be replicated across multiple clinicians and settings. Despite these limitations, the study clearly demonstrates that there is an important gap in the literature regarding the way in which individuals self-report their symptoms depending on the context in which they are asked [12]. Further research is necessary to better understand the influence of syndrome context on the perception and self-reporting of symptoms and to identify any biases that may impact the accuracy of self-reported symptoms.

The aim of this study was to examine the diversity of self-reported fatigue symptom severity in MDD compared to GAD. The conclusions are given more confidence using statistical tests, and the sample size of 100 patients is adequate to produce accurate results. The use of self-reported metrics and the retrospective nature of the study, however, raise the possibility of biases and mistakes in the data [13]. The authors are unable to comment on the diversity of the study population as demographic data was not collected due to it not being part of the IRB approval obtained. Additionally, the study does not investigate any potential underlying mechanisms that might be responsible for the observed variations in the degree of self-reported fatigue symptoms. Overall, this study offers insightful information about the significance of considering symptom reporting context in diagnostic and treatment plans for people with MDD and GAD, but further research is required to completely comprehend the complexity of this issue.

## Conclusions

These findings suggest that self-reported fatigue may vary depending on the context of the syndrome in which it is assessed. Future research is necessary to replicate these findings, expand the scope of the study, and examine the impact of the order of symptom severity reporting on other self-reported symptoms. Mental health professionals should be aware of the influence of the order of questionnaire administration on self-reported symptom severity in their clinical practices. Future research should be done by collecting demographic information from the same patient charts and analyzing the resultant data to see if there are differences based on age, sex, gender, ethnicity, etc.

These findings have important implications for the clinical assessment and treatment of individuals with MDD and GAD. It is established in the DSM-5 criteria that fatigue is a common symptom experienced by individuals with both MDD and GAD. However, this study suggests that the way in which fatigue is perceived and self-reported may vary based on the syndrome in which it is assessed. Understanding the effect of syndrome context on self-reported ratings of fatigue can help healthcare providers provide more accurate and effective treatment for their patients.
